# Supplementary material for: Dual Inhibition of HIV-1 and Cathepsin L Proteases by Sarcandra glabra
Source: Molecules. 2022 Aug 29;27(17):5552. doi: 10.3390/molecules27175552 (PMC9457736; doi:10.3390/molecules27175552)
Supplement: Supplementary file 1 [file molecules-27-05552-s001.zip › molecules-1852624-supplementary.pdf]

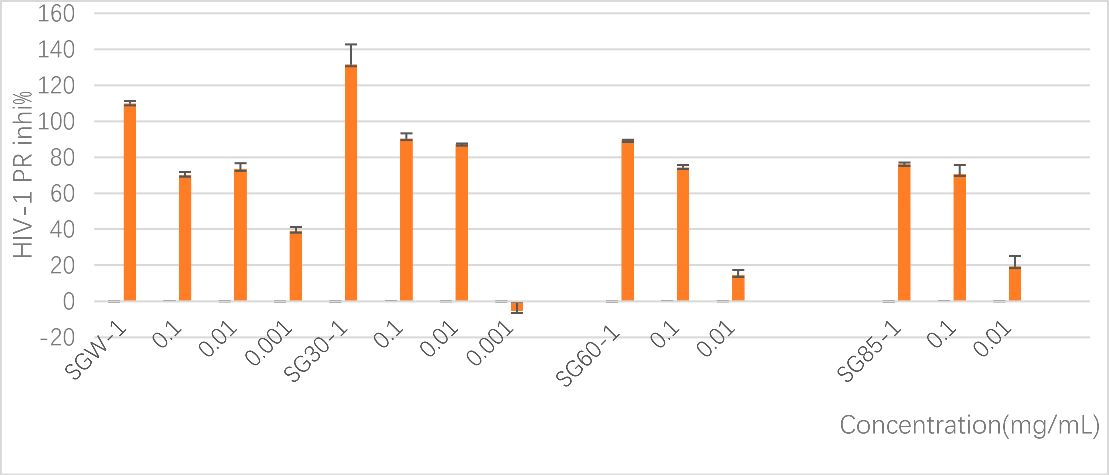

(**a**) Four extracts of *Sarcandra glabra* against HIV-1 PR (n = 3)

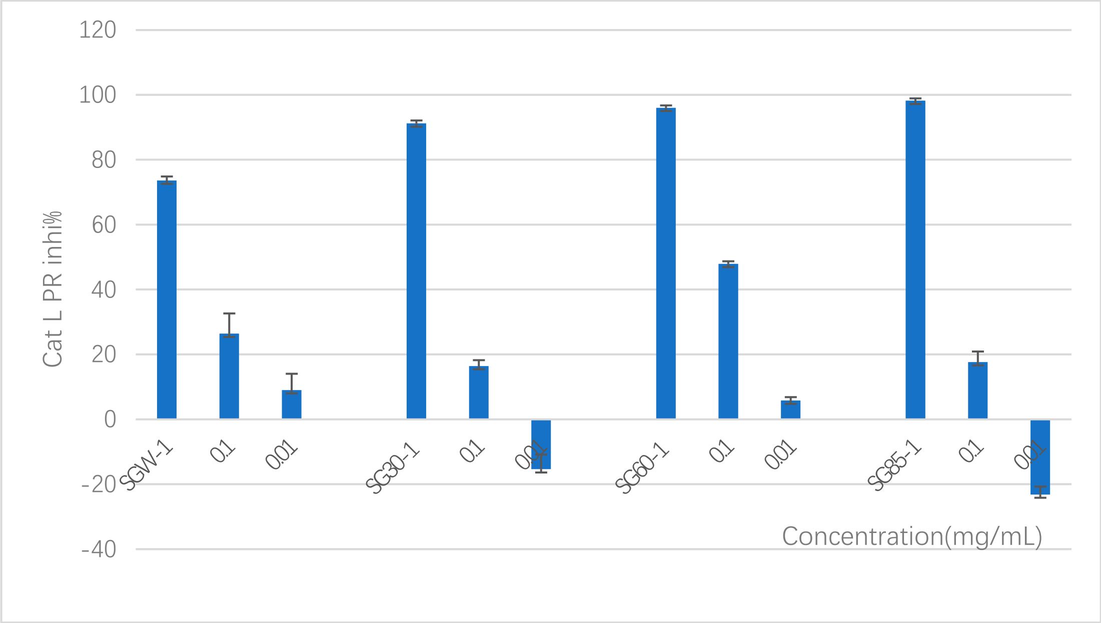

(**b**) Four extracts of *Sarcandra glabra* against Cat L PR (n = 3)

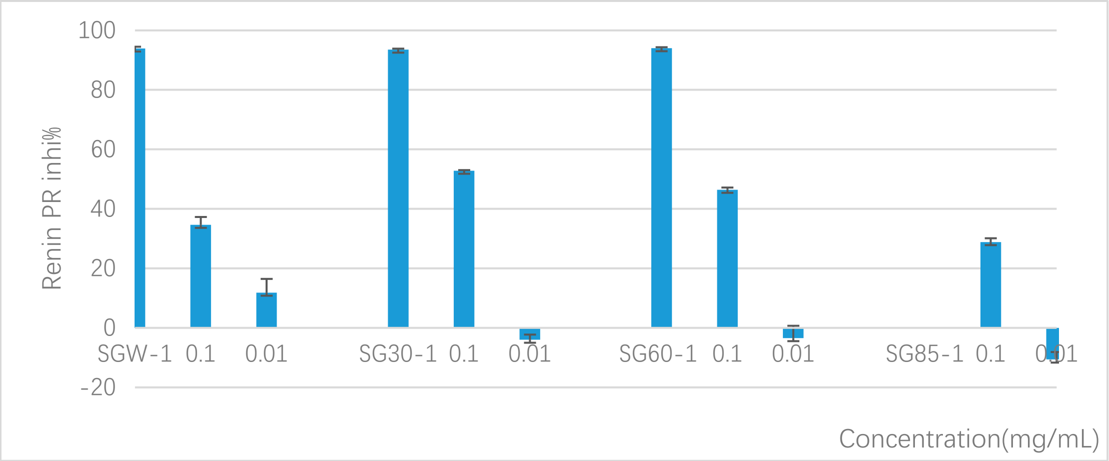

(**c**) Four extracts of *Sarcandra glabra* against Renin PR (n = 3)

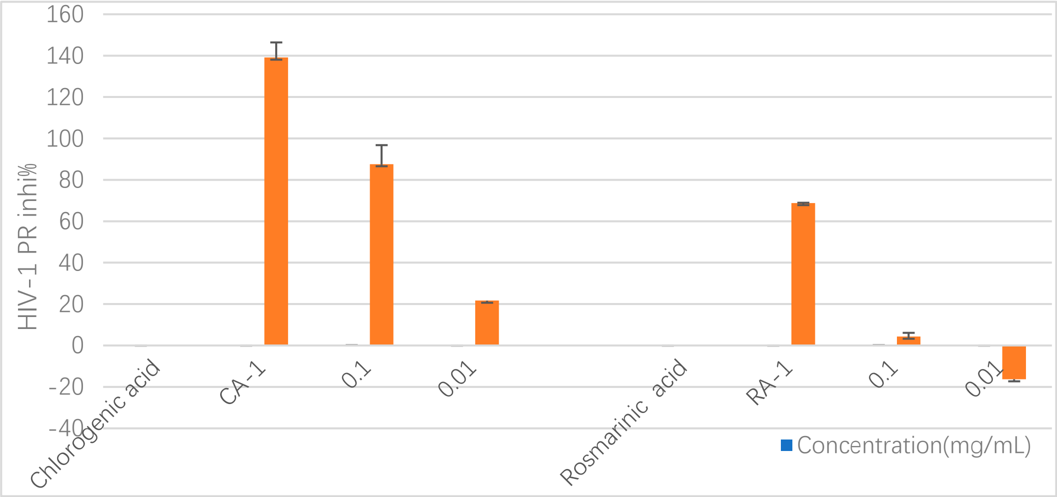

(**d**) Chlorogenic acid and Rosmarinic acid inhibit HIV-1 PR (*n* = 3)

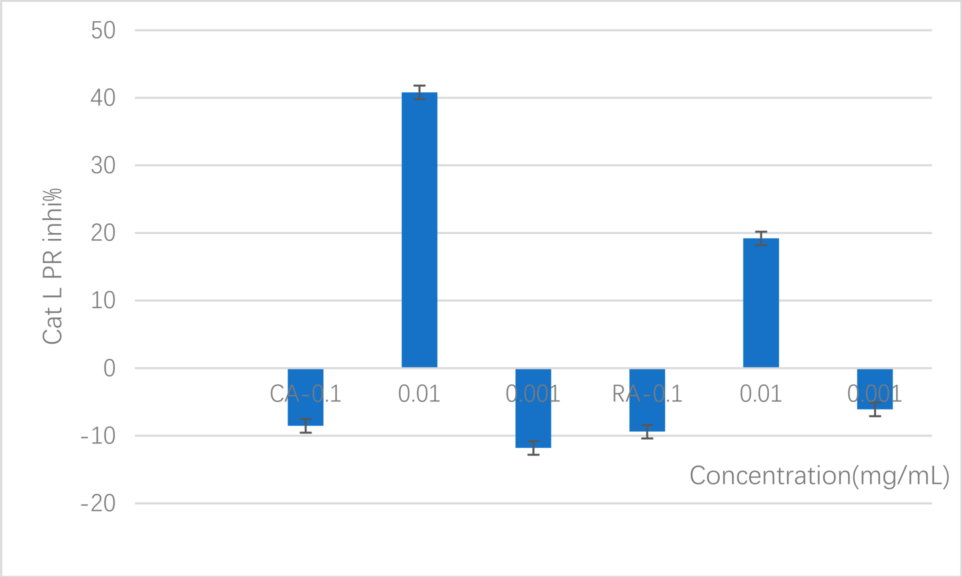

(**e**) Chlorogenic acid and Rosmarinic acid inhibit Cat L PR (*n* = 3)

Figure S1. Four extracts and main ingredients anti-HIV (**a**–**c**) and cathepsin L protease inhibition (**d**,**e**).

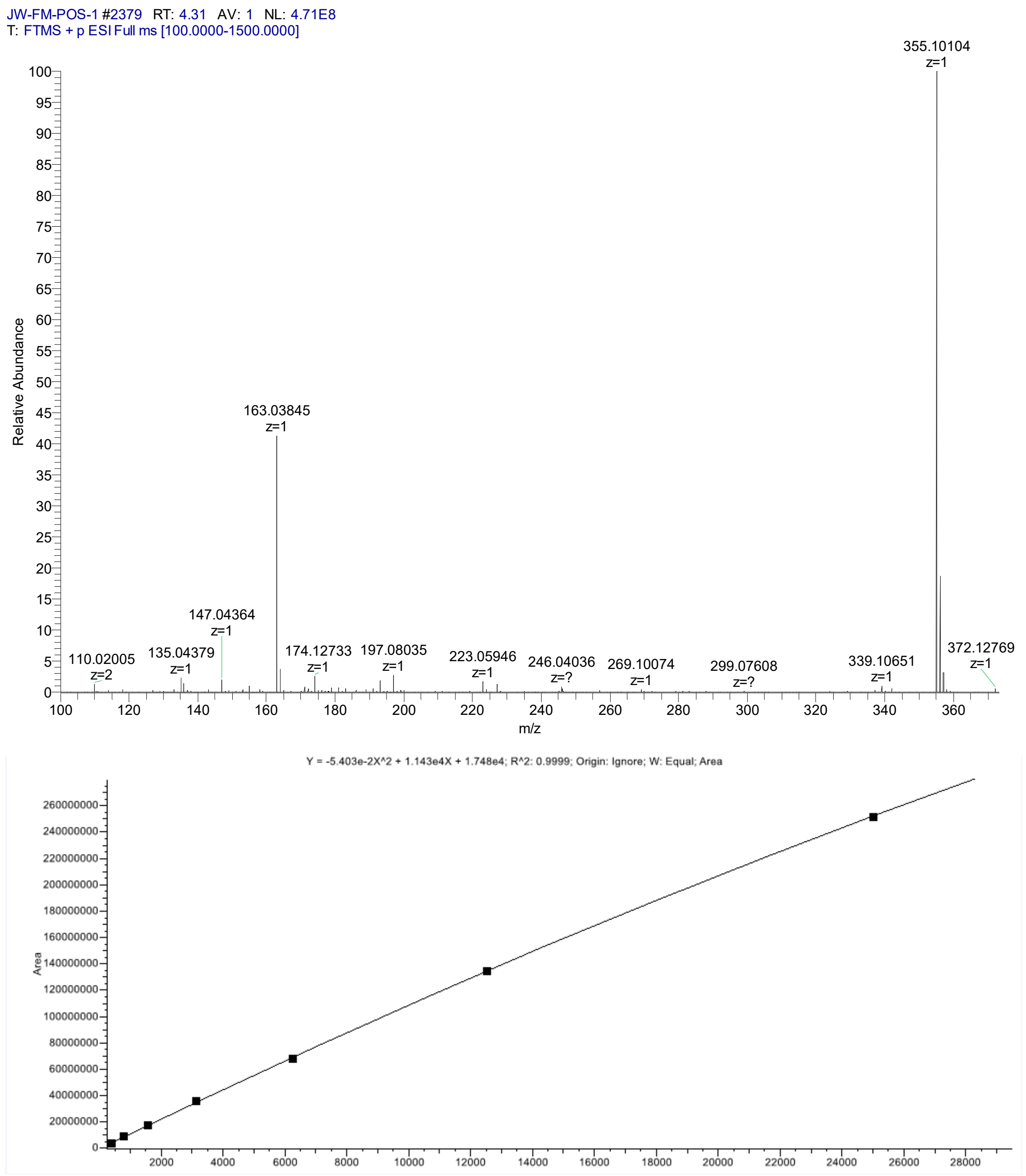

Figure S2. MS Spectrogram and Quantitative Curve of chlorogenic acid.
